# First identification of orf virus from an outbreak of contagious ecthyma in Polish sheep

**DOI:** 10.2478/jvetres-2026-0035

**Published:** 2026-06-30

**Authors:** Magdalena Larska, Agnieszka Nowakowska, Urszula Pękala-Duda

**Affiliations:** 1 Department of Virology and Viral Animal Diseases, National Veterinary Research Institute, 24-100 Puławy, Poland; 2 KARMEL Veterinary Clinic, 46-250 Wółczyn, Poland

**Keywords:** orf virus, parapoxvirus, sheep

## Abstract

**Introduction:**

Contagious ecthyma (CE) is a skin disease of goats, sheep and some other domesticated and wild ruminants worldwide, including Poland. The infection is caused by the orf virus (ORFV) of the *Parapoxvirus* genus. It is characterised by the appearance of skin lesions in the form of papules and pustules, most often in the area of the mouth and nostrils, hooves and genitals. The disease causes economic losses, especially in countries with high sheep and goat production. In Europe, the threat posed by this disease is rather underestimated, but following the re-emergence of foot-and-mouth disease (FMD) in the region in 2025, there is increased vigilance and a need to have differential diagnostic methods proven and ready.

**Material and Methods:**

DNA was extracted from lesions on mouth and hooves of sheep. The animals were on a farm located in southwestern Poland on which FMD was ruled out. A PCR was performed using two pairs of specific primers for detecting *vIL10* and *GIF* gene fragments which were then sequenced.

**Results:**

Sequencing confirmed ORFV infection in all tested sheep samples. The sequences obtained for Polish viruses were also highly homologous to other parapoxviruses, indicating that the method used may be applicable for broad diagnosis of parapoxvirus infections not only in ruminants.

**Conclusion:**

This is the first molecular characterisation of ORFV in sheep in Poland. It signals a potential herd biosecurity problem in view of the neglected but re-emerging nature of CE. The marked genetic divergence between Polish field ORFV isolates and currently available vaccine strains may influence CE vaccine efficacy in Poland.

## Introduction

Contagious ecthyma (CE), sore mouth or contagious pustular dermatitis is a highly contagious viral skin disease which occurs worldwide, both in pastures and in feedlots. It is regarded as a neglected disease as it is perceived as mild and is underreported. However, intensification of animal production, combined with negligence in the application of biosecurity measures contribute to the re-emergence of the disease. These factors are exacerbated by insufficient diagnostic capacity to raise its zoonotic potential, even in highly developed countries (7, 19, 27). Contagious ecthyma primarily affects sheep and goats, but cases of infection have also been reported in other domestic and wild ruminants and mammals. Cases are reported more frequently in late summer, autumn and winter (28). The aetiological agent of contagious ecthyma is the orf virus (ORFV), a member of the *Parapoxvirus* genus and the Poxviridae family. The orf virus is antigenically and genetically related to other parapoxviruses including bovine papular stomatitis virus, red deer parapoxvirus New Zealand pseudocowpox virus and sealpox virus, as well as to the tentative genus members Auzdik disease (camelpox) virus and chamois contagious ecthyma virus. The ORFV virion is a large, ovoid-shaped, lipid enveloped particle containing a linear, double-stranded DNA genome (130–140 kb) with conserved central and variable inverted terminal repeats involved in virulence and pathogenesis, enveloped in a complex protein structure organised into a distinctive tubular or threadlike appearance. Parapoxvirus genomes encode some proteins of high immunomodulatory potential, such as viral interleukin 10 (vIL-10, also termed ORFV127), granulocytemacrophage colony stimulating factor/interleukin 2 (GM-CSF/IL-2) inhibitory protein (GIF/ORFV117) or orf virus interferon resistance protein (OVIFNR/ORFV020) (14, 16, 39). The host specificity and immunoregulatory capacity of parapoxviruses have been used in vaccine developments and therapeutic strategies (24, 36, 39).

Although, the clinical symptoms of CE are very characteristic, laboratory diagnosis is necessary to confirm the presence of orf virus. Contagious ecthyma symptoms are observed in the skin around the mouth and nostrils and less frequently on the cheeks. Initially, they appear as papules that gradually develop into vesicles and pustules, which then develop into thick crusts. Less frequently, similar lesions affect the hooves and the anus and genitals (vagina and prepuce) (18). In nursing mothers, skin lesions may also be observed on the mammary glands, which causes problems with suckling in young animals. If secondary lesions appear because of bacterial contamination, the lesions are milder and resolve more quickly, usually within 2–3 weeks. Occasionally, secondary infections may evolve into gastroenteritis or bronchopneumonia. Morbidity from this disease can reach up to 100% in a herd and mortality due to secondary bacterial infections can reach 15% (34). Orf virus is sensitive to high and very low temperatures, humidity and UV radiation, as well as chloroform, benzene and toluene. In a cool, dry and shaded environment (in wool, hides and in animal bedding and on farm equipment), it can survive for several months and it can remain infectious even up to 17 years at room temperature (34). Contagious ecthyma is highly contagious and numerous cases of orf virus infection have been reported in humans from various parts of the world, particularly among shepherds, farmers, veterinarians, butchers and immunocompromised individuals (2, 19). The symptoms include skin lesions limited to the exposed parts of the body, accompanied by symptoms such as pain, itching, and less frequently, fever or malaise. The lesions usually heal spontaneously within 3–6 weeks. Orf virus is a global pathogen that causes significant losses in livestock production; however, the impact is probably underestimated as it is not a notifiable disease. Lesions caused by it threaten optimal productivity, reduce the market value of meat, hide and wool, and disrupt domestic and international trade in animals and animal products.

## Material and Methods

### Sample collection

**Fig. 1.**
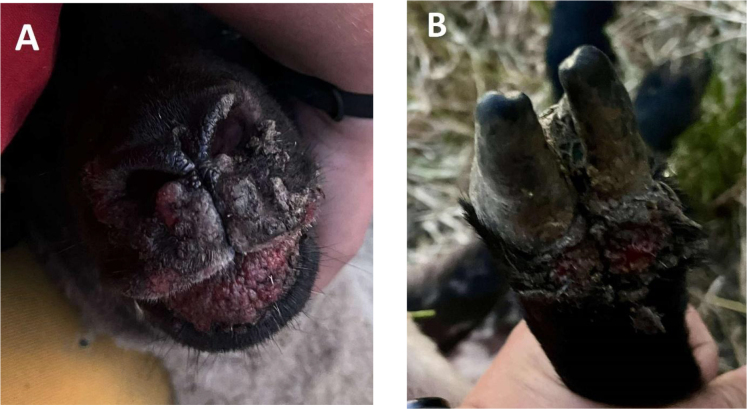
Erythema, pustules and crusts on the muzzle (A) and papules at the coronary band of the front feet (B) in Polish Blackhead sheep infected with orf virus

The material originated from a sheep flock in the southwestern part of Poland (Kluczbork County in Opole Voivodeship), which displayed signs of vesicular disease. The current situation of foot-and-mouth disease (FMD) occurrence in Europe (35) caused this disease to be suspected first; however, this was discounted by the diagnostic RT-PCR at the FMD reference laboratory at the National Veterinary Research Institute in Zduńska Wola, Poland. The herd consisted of 28 Blackhead sheep and the symptoms were observed in 8 (28.6%) individuals aged 1–2 years. First, the sheep displayed symptoms of erythema and pustules with serous discharge in the area of the mouth, nostrils and coronary bands, which became purulent after a few days. Lesions were noted which were covered with crusting (Fig. 1). The animals had difficulties in feeding because of painful lesions on the lips; however, no lameness or other symptoms were reported by the breeder. Samples consisted of skin scrapings from the area of the mouth and hooves collected from four of these sheep in July 2025.

### Sample preparation

Preparation began with extraction of DNA from homogenised scrapings of diseased skin of the mouth and hooves. A series of 10% homogenates were prepared in Eagle’s MEM (Sigma-Aldrich, St. Louis, MO, USA) using tubes containing 1.4-mm diameter ceramic (zirconium silicate) beads (Lysing Matrix D, MP Biomedicals, Irvine, CA, USA). The mixture was homogenised using a Fastprep-24 5G homogeniser (MP Biomedicals) for 45 s at 4.5 m/s. Viral DNA was extracted from 200 μL of supernatant using a QIAamp DNA Mini kit (Qiagen, Hilden, Germany) according to the manufacturer’s guidelines. It was resuspended in 60 μL of an elution buffer and stored at -20°C until testing.

### PCR

Two specific pairs of primers for parapoxvirus were used for PCR reactions: GIF5 (5’-GCT CTA GGA AAG ATG GCG TG-% and GIF6 (5’-GTA CTC CTG GCT GAA GAG CG-3’) for a 408-bp GM-CSF-and IL-2-inhibitory factor (GIF) gene fragment, and vIL-10-3 (5’-ATG CTA CTC ACA CAG TCG CTC C-3’) and vIL-10-4 (5’-TAT GTC GAA CTC GCT CAT GGC C-3’) for a 300-bp putative interleukin-10 (IL-10) homologue gene (22). The 25-μL reaction mix used was composed of 17.5 μL of water, 2.5 μL of 10× Buffer for AccuTaq LA (long and accurate) DNA Polymerase (Sigma-Aldrich), 0.5 μL of JumpStart AccuTaq LA DNA Polymerase Mix (2.5 U/mL) (Sigma-Aldrich), 0.5 μL of dNTP mix (ThermoFisher Scientific, Carlsbad, CA, USA), 1 μL (10 μM) each of GIF5 and GIF6 or vIL-10-3 and vIL-10-4 and 2 μL of DNA. The reaction conditions were 15 min of incubation at 95°C; 5 cycles of amplification, each consisting of 30 s of denaturation at 94°C, 2 min of primer annealing (for both primer pairs) at 57°C and 30 s of elongation at 72°C; 35 cycles of amplification, each consisting of 30 s of denaturation at 94°C, 30 s of annealing at 57°C (for GIF5/GIF6 and vIL-10-3/vIL-10-4) and 30 s of elongation at 72°C; and 10 min of final elongation at 72°C. A 5-μL aliquot of the PCR product was used for visualisation by electrophoresis on 1.5% agarose gel with GeneRuler Low-Range DNA Ladder for molecular size marking (Cat. No. SM1193, ThermoFisher Scientific). A sample was regarded as positive if the expected 408-bp band (for GIF5 and GIF6) or 300-bp band (for vIL-10-3 and vIL-10-4) was visible on the gel. All the positive PCR products were sequenced using the Sanger method (performed by Genomed, Warsaw, Poland). The obtained nucleotide sequences were compared with the sequences retrieved from GenBank according to their sequence homology using BLASTn (NIH) (4, 6).

## Results

**Fig. 2.**
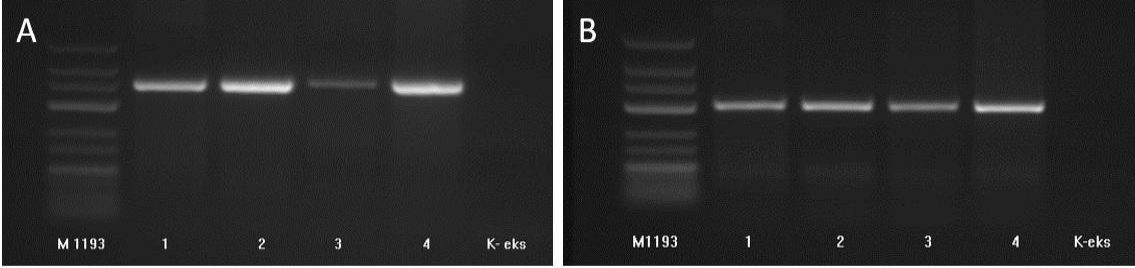
Amplification of orf virus genetic material from skin scraping samples from infected Polish Blackhead sheep. Analysis of PCR products with GIF5 and GIF6 primers with visible 408-bp amplicon (A), and with vIL03 and vIL04 primers with visible 300-bp amplicon (B). M1193 – molecular size marker; K-eks – no template control

All four samples tested for orf virus were PCR positive, showing a predicted PCR amplicon size of 408 bp for GIF5/GIF6 (Fig. 2a) and 300 bp for vIL03/vIL04 (Fig. 2b).

High homology (almost 98% sequence identity) of nucleotide sequences of the amplicons of the GIF5/GIF6 and vIL03/vIL04 primers with respective GenBank reference sequences MN454854 and LR594616 allowed the virus to be identified as orf virus. The sequences within the two genes obtained from samples from all four sheep showed 100% homology (Tables 1 and 2). However, while *GIF* sequences matched sequences found to be highly conserved between different parapoxviruses from very different hosts (cattle, walrus, muskox, dromedary camel, seal and Japanese serow) (Table 1), the variation in the analysed *vIL-10* sequences was higher, maintaining homology only within orf virus isolates from sheep and goats (Table 2). A greater similarity of orf virus to the pseudocowpox virus was additionally found in the *vIL-10* gene. The remaining sequences originating from camels, bats, seals and macaques or another virus species like bovine papular stomatitis virus varied more, but these differences did not exceed 5%, which corresponded to approximately 15 nucleotides (Table 2). Importantly, while *GIF* sequences of Polish isolates showed 100% homology with the NZ2 reference orf virus strain and ORFV-1 and D1701 vaccine strains (Table 1), they differed significantly (94.2–94.5%) from these strains in terms of the *vIL-10* gene (Table 2).

**Table 1.** Similarity matrix sequence heatmap for pairwise amino acid identity of granulocyte-macrophage colony-stimulating factor gene fragments of parapoxviruses detected in sheep in Kluczbork, Poland and other sequences retrieved from GenBank (these preceded by their accession Nos). The colour intensity of the grid indicates degree of similarity (darker for high and lighter for lower)

	PQ374835.1_ORFV-1V	PP565901.1_D1701V	ON805832.1	MF497783.1	MF175205.1	KM057389.1	KF666566.1	GU460372.1	AY605983.1	AY605975.1	OR452360.1	ON805831.1	LC476575.1	AY605988.1_NZ2	orf_virus_1_Poland_2025	orf_virus_2_Poland_2025	orf_virus_3_Poland_2025	orf_virus_4_Poland_2025
PQ374835.1_orf_virus_ORFV-1V2024_vaccine	100																	
PP565901.1_orf_virus_D1701_vaccine	100	100																
ON805832.1_orf_virus_NAV_sheep_Spain_2018	100	100	100															
MF497783.1_pseudocowpox_virus_Iraq-D.NO1_cattle_Iraq_2017	99.9	99.9	99.9	100														
MF175205.1_parapoxvirus_sp._O.r.r._Svalbard_atlantic_walrus_Norway_2015	100	100	100	99.9	100													
KM057389.1_orf_virus_2010-147_Muskox_USA(Alaska)_2010	100	100	100	99.9	100	100												
KF666566.1_orf_virus_Xinjiang2_goat_China_2013	99.9	100	99.9	99.9	99.9	99.9	100											
GU460372.1_orf_virus_Cam09_dromedary_camel_India_2008	99.9	99.9	99.9	99.9	99.9	99.9	99.9	100										
AY605983.1_pseudocowpox_virus_N71.2Bos_cattle_Norway	100	100	100	99.9	100	100	99.9	99.9	100									
AY605975.1_seal_parapoxvirus_Sc95.1Hg_grey_seal_United_Kingdom	99.9	100	100	99.9	100	100	99.9	99.9	100	100								
OR452360.1_orf_virus_ORFVGoatBangladeshBLRI-012021_goat_Bangladesh_2021	100	100	100	99.9	100	100	100	99.9	100	99.9	100							
ON805831.1_orf_virus_HRE_sheep_Argentina_2018	100	100	100	99.9	100	100	99.9	99.9	100	100	100	100						
LC476575.1_orf_virus_S-1_japanese_serow_Japan_1985	100	100	100	100	100	100	100	99.9	100	100	100	100	100					
AY605988.1_orf_virus_NZ2_New_Zealand	99.9	99.9	100	99.9	100	100	99.9	99.9	100	100	100	100	100	100				
orf_virus_isolate_kluczbork_1_sheep_Poland_2025	100	100	100	99.9	100	100	99.9	99.9	100	100	99.9	100	100	100	100			
orf_virus_isolate_kluczbork_2_sheep_Poland_2025	100	100	100	99.9	100	100	99.9	99.9	100	100	99.9	100	100	100	100	100		
orf_virus_isolate_kluczbork_3_sheep_Poland_2025	100	100	100	99.9	100	100	99.9	99.9	100	100	99.9	100	100	100	100	100	100	
orf_virus_isolate_kluczbork_4_sheep_Poland_2025	100	100	100	99.9	100	100	99.9	99.9	100	100	99.9	100	100	100	100	100	100	100

**Table 2.** Similarity matrix sequence heatmap for pairwise amino acid identity of viral interleukin 10 orthologue gene fragments of parapoxviruses detected in sheep in Kluczbork, Poland and other sequences retrieved from GenBank (these preceded by their accession Nos). The colour intensity of the grid indicates degree of similarity (darker for high and lighter for lower)

	PQ374835.1_ORFV-1V	PP565901.1_D1701V	XM_005885225.2	OR900038.1	KY382358.2	AY606011.1	KF927101.1	KM098083.1	KM098071.7	NC_055142.1	PP943426.1	KM875472.1	JQ728421.1	AY606006.1_NZ2	orf_virus_1_Poland_2025	orf_virus_2_Poland_2025	orf_virus_3_Poland_2025	orf_virus_4_Poland_2025
PQ374835.1_orf_virus_isolate_ORFV-1V2024_vaccine	100																	
PP565901.1_orf_virus_isolate_D1701_vaccine	100	100																
XM_005885225.2_brandts_bat_Russia_2011	94.2	94.5	100															
OR900038.1_sheep_isolate_1_Egypt_2023	94.2	94.5	99.7	100														
KY382358.2_seal_parapoxvirus_isolate_AFK76s1_seal_Poland_2015	94.4	94.1	97.6	97.4	100													
AY606011.1_pseudocowpox_virus_isolate_No79.1Bos_cattle_Norway	94.1	94.3	98.3	98.2	97.7	100												
KF927101.1_orf_virus_isolate_SV269_11_sheep_Brazil_2011	94.2	94.3	98.3	98.2	97.8	100	100											
KM098083.1_orf_virus_isolate_2011-106_Dalls_sheep_USA_2011	94.1	94.3	98.3	98.2	97.7	100	100	100										
KM098071.7_orf_virus_isolate_2005-167_mountain_goat_USA_2005	94.1	94.3	98.3	98.2	97.7	100	100	100	100									
NC_055142.1_lymphocryptovirus_macaca/pfe-lcl-E3_long-tailed_macaque_USA_2012	93.9	94	98.1	97.7	97.2	98.3	98.3	98.3	98.3	100								
PP943426.1_orf_virus_isolate_FX17_goat_China_2012	94.1	94.2	98.3	98.2	97.7	100	100	100	100	98.1	100							
KM875472.1_bovine_papular_stomatitis_virus_strain_BV-TX09c1_cattle_USA_2009	94.5	94.7	98.6	98.7	98.1	98.6	98.6	98.6	98.6	97.9	98.6	100						
JQ728421.1_pseudocowpox_virus_dromedary_camel_India_2011	92.9	93.1	94.2	93.7	93.2	93.5	93.5	93.5	93.5	93.6	93.6	93	100					
AY606006.1_orf_virus_isolate_NZ2_NewZealand	100	100	94.3	94.4	94.1	94.1	94.2	94.1	94.1	94	94	94.7	92.9	100				
orf_virus_isolate_kluczbork_1_sheep_Poland_2025	94.3	94.5	98.3	98.3	97.8	100	100	100	100	98.2	100	98.6	93.4	94.3	100			
orf_virus_isolate_kluczbork_2_sheep_Poland_2025	94.2	94.4	98.3	98.2	97.7	100	100	100	100	98.2	100	98.6	93.5	94.2	100	100		
orf_virus_isolate_kluczbork_3_sheep_Poland_2025	94.2	94.4	98.3	98.2	97.7	100	100	100	100	98.2	100	98.6	93.5	94.2	100	100	100	
orf_virus_isolate_kluczbork_4_sheep_Poland_2025	94.3	94.5	98.3	98.3	97.8	100	100	100	100	98.2	100	98.6	93.4	94.3	100	100	100	100

**Fig. 3.**
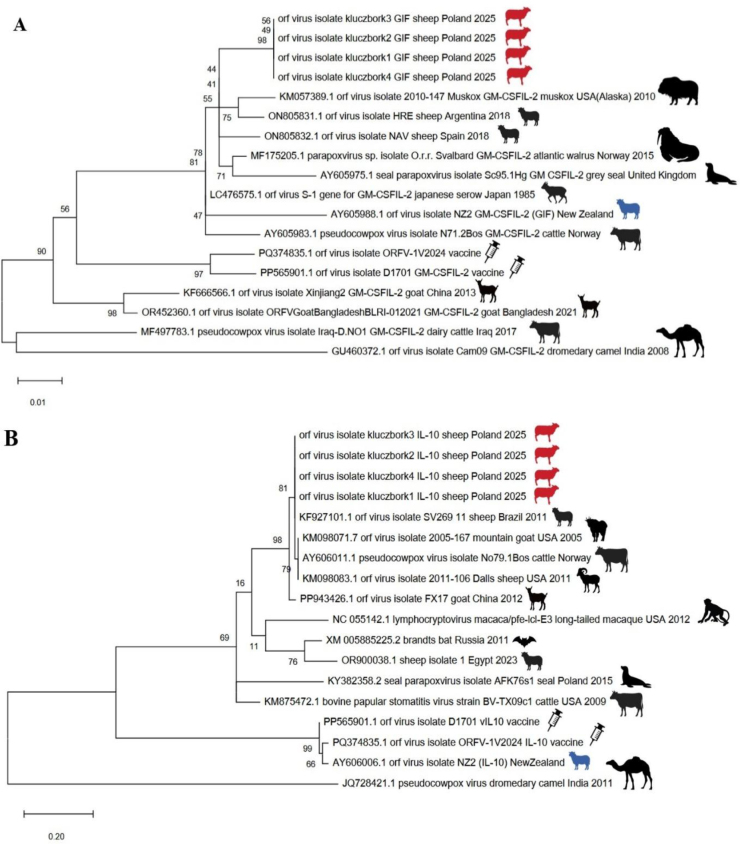
Maximum-likelihood trees based on the nucleotide sequences of the granulocyte-macrophage colony-stimulating factor and interleukin-2 inhibition factor (*GIF*) (A) and viral interleukin 10 orthologue (*vIL-10*) (B) gene fragments of parapoxviruses detected in sheep in Kluczbork, Poland (sheep in red), Orf virus reference strain NZ2 (sheep in blue), two vaccine strains (marked with syringes) and other sequences retrieved from GenBank (all strains not isolated in the present research preceded by their accession Nos). Bootstrap values were generated for 1,000 repeats

Phylogenetic trees created using these sequences confirmed that within the *GIF* gene, the Polish strain of the orf virus showed greater similarity to European reference strains of sheep orf virus and of other parapoxviruses of marine mammals than to orf viruses from Asia or South America (Fig. 3A). However, the relationship based on the *vIL-10* gene was more host specific, and the sequences of the Polish orf virus grouped with the reference sequences from ruminants (Fig. 3B).

## Discussion

Despite being identified in sheep as early as the 18^th^ century, CE remains a neglected disease both in terms of epidemiology and diagnostic capabilities. The neglect is exemplified by its non-inclusion on the World Organisation for Animal Health notifiable disease list (9, 10, 19, 31). However, it occurs worldwide and impacts any country proportionately to the size of its small ruminant production sector (3). It is curious that reports of orf virus transmission to humans prevail in Europe (1, 21, 20, 27, 38), while data on CE occurrence in reservoir species such as sheep and goats is lacking. The available evidence is insufficient to characterise orf virus prevalence across Europe broadly. The single identified study was geographically limited to the UK, relied on questionnaire-based data collection and did not specify diagnostic methods for case identification (28). This study revealed a substantial disparity in orf prevalence between age groups. Lambs exhibited a prevalence of 19.53%, approximately ten times higher than the 1.88% prevalence observed in ewes. Vaccination had some protective effects especially in lambs; however, lower efficiency of vaccinations was reported, which was theorised to result from the suspected incorrect administration by UK farmers (33).

The most recent research on the occurrence of orf virus infections in Poland dates back almost four decades (30). Before this, orf virus was considered among the possible aetiologies of a mouth and upper respiratory-tract disease in sheep imported into Poland from Romania in 1952. Chodkowski and Żebrowski (8) demonstrated that the condition was caused by a transmissible agent. However, the relatively high mortality (15%); high fever (42°C); emaciation of the body; the severity of the lesions including diphtheroid-ulcerative and gangrenous inflammation of the gums, tongue and palate; pneumonia, serofibrinous pleurisy, septicaemia; and enteritidis suggested a different disease to CE.

Another report described an infection with the viral agent of CE in a man in 1956 (23). The orf virus was visualised for the first time using electron microscopy at the National Veterinary Research Institute in Puławy in 1978 (11), and isolated in primary sheep kidney cultures from scabs of sheep originating from the then Chełm and Lublin provinces in 1989 (30). The only parapoxvirus genetically characterised so far is the seal parapoxvirus (SePPV) (GenBank No. KY382358), which was recovered from a grey seal (*Halichoerus grypus*) at the rehabilitation centre in Hel, Poland (17). Work on the disease was not continued. Data on the potential impact of CE on sheep and goat farming in the country, where the livestock population numbers approximately 270,000 and 64,000, respectively, are lacking. Our study introduces molecular methodology for orf virus detection. The changes observed in Polish sheep corresponded to those observed in infection with the orf virus elsewhere; however, as they are not specific to infection with a single pathogen (Table 4), the identification of the aetiological agent of the disease causing them is crucial. An epidemiological investigation indicated the possibility of the virus having been introduced with new sheep that had arrived shortly before the July 2025 sampling. Around that time, the sheep were sheared with equipment owned by the external shearing contractor. No such cases had been reported in the flock previously, so it is reasonable to assume that the virus infection occurred as a result of one of these events. This emphasises the significance of applying appropriate biosecurity measures.

**Table 4.** Differential diagnoses of sheep infectious diseases involving changes in the mouth, hooves, teats or external reproductive organs

Feature	Contagious ecthyma	Sheep pox	Foot-and-mouth disease	Bluetongue	Peste des petits ruminants
Causative agent	Orf virus (a DNA virus in the *Parapoxvirus* genus).	Sheeppox virus (a DNA virus in the *Capripoxvirus* genus).	Foot-and-mouth disease virus (an RNA virus in the *Aphthovirus* genus).	Bluetongue virus (an RNA virus in the *Orbivirus* genus).	Peste des petits ruminants virus (an RNA virus in the *Morbillivirus* genus).
Affected species	Sheep, goats and wild ruminants (zoonotic to humans).	Primarily sheep, but also goats (goatpox).	All cloven-hoofed animals (cattle, sheep, goats, pigs).	Sheep, goats, cattle and wild ruminants.	Primarily sheep and goats.
Clinical signs	Localised, proliferative skin lesions/scabs, mainly around the mouth and lips of young animals; heals without scarring in 4–5 weeks.	Severe, generalised pox lesions throughout the skin and mucous membranes, persistent fever, pneumonia and high mortality.	Fever, vesicular lesions (blisters) and erosions in the mouth (especially tongue), muzzle, feet and teats; causes lameness.	Oedema of the face/muzzle, soreness of the mouth and feet, oral ulcers, lameness and general weakness.	High fever, severe nasal/ocular discharge (which becomes purulent), diarrhoea, mouth lesions and bronchopneumonia.
Affected systems	Integumentary	Integumentary and respiratory	Epithelial surfaces	Multiple systems, including oral mucosa and circulatory system	Respiratory and gastrointestinal
Transmission	Direct contact with infected animals or contaminated environment.	Direct contact and possibly insects.	Direct contact, aerosols and contaminated fomites.	Vector-borne by *Culicoides* biting midges.	Direct contact and contaminated feed/water.
Mortality rate	Low (unless secondary infection or starvation in lambs).	Can be very high (up to 100% in lambs).	Low mortality in adult sheep; production losses are the main impact.	Variable, but can be high, especially in lambs.	Can be very high (up to 100% in outbreaks).
Zoonotic potential	Yes	No	No	No	No
WOAH notifiable disease	No	Yes	Yes	Yes	Yes
Present	Endemic worldwide	Africa, the Middle Eastand Asia^*^	Asia, Africa and South America	Widespread globally	Africa, the Middle East and Asia^*^

* – increased risk for Europe in recent years

Our study was was undertaken in response to the need to introduce a diagnostic method as a tool for outbreak control, and to differentiate CE from more severe conditions, avoid misdiagnosis and prevent unnecessary treatments such as antibiotic administration. This need has grown with the emergence of new epidemiologic threats such as FMD, bluetongue or peste des petits ruminants, which may be mimicked by orf virus infection (Table 4) (5, 13, 25, 26, 35). We aimed to develop a rapid orf virus identification method also to help prevent its zoonotic spread (animal to human), especially since there are no specific treatments or human vaccinations available in the country and early management is consequently highly advantageous. The studied outbreak coincided with the re-emergence of FMD in Europe (35) and incursion of BTV-3 into Poland (25).

Our main goal was not to perform a phylogenetic analysis of the Polish strains, but to evaluate the selected primers in terms of suitability in a PCR for diagnostic purposes. Both pairs, as used by Klein and Tryland (22), allowed the virus to be detected in all samples, confirming that they can be used for diagnostic purposes and for surveillance. In our hands, the *vIL-10* gene could additionally be used for epidemiological investigations taking advantage of its variability; this will be the subject of further research. Because we only included parapoxviruses which originated from different hosts from different regions in the phylogenetic analysis, our findings may differ from previous ones (32). In general, the *vIL-10* gene (ORF127) is highly conserved and is believed to be a recent evolutionary acquisition by horizontal gene transfer from a mammalian host (15). Contrastingly, the *GIF* gene (ORF117) shows high genetic variability, which allows adaptation to the specific cytokines of different host species, as the binding specificity needs to evolve for the host’s unique receptor structures (12). This gene does not encode proteins resembling any known mammalian GM-CSF or IL-2 binding proteins, which indicates a unique evolutionary path of the gene.

The method used had previously been employed in the diagnosis of parapoxviruses in reindeer (22, 37); therefore, it will probably also be applied in research on cervids, especially in closed breeding facilities, or in endangered species of European bison. We believe that one of the most valuable observations is the large genetic divergence between Polish orf virus strains and the NZ6 reference strain from New Zealand, and even more so between it and the ORFV-1F (GO-BT) and D1701 vaccine strains (29). Varying orf virus vaccine efficacy was observed by Zhao *et al*. (40). This inconsistency in protection resulted from strain differences, whereby live vaccines sometimes failed to provide broad protection against diverse field strains, leading to outbreaks even in vaccinated flocks. The findings made by Zhao *et al*. (40). draw attention to the genetic mismatch and short-lived immunity issues in vaccination against contagious ecthyma In order to protect animals, it seems necessary to review the strains used for the production of CE vaccines in Europe.

## Conclusion

The introduced PCR facilitated the confirmation of the first outbreak of contagious ecthyma. The amplified fragments of orf virus showed relatively low variability, which allows them to be used for the differentiation of various parapoxviruses. In addition, they allowed the determination of phylogenetic relationships, which may facilitate epidemiological investigations. Polish ORFV strains differed quite significantly from the reference strain and strains used in commercial vaccines, which may affect the efficiency of vaccination against CE in the country. These findings may have important implications for molecular surveillance and future vaccine development against contagious ecthyma in Europe.
